# The transcription factor Sox10 is an essential determinant of branching morphogenesis and involution in the mouse mammary gland

**DOI:** 10.1038/s41598-020-74664-y

**Published:** 2020-10-20

**Authors:** Svenja Mertelmeyer, Matthias Weider, Tina Baroti, Simone Reiprich, Franziska Fröb, C. Claus Stolt, Kay-Uwe Wagner, Michael Wegner

**Affiliations:** 1 Institut für Biochemie, Emil-Fischer-Zentrum, Friedrich-Alexander-Universität Erlangen-Nürnberg, Fahrstrasse 17, 91054 Erlangen, Germany; 2 Barbara Ann Karmanos Cancer Institute, Detroit, USA; 3 Wayne State University School of Medicine, Detroit, MI USA; 4 Zahnklinik 3 – Kieferorthopädie, Universitätsklinikum Erlangen, FAU Erlangen-Nürnberg, Glückstrasse 6, 91054 Erlangen, Germany

**Keywords:** Developmental biology, Differentiation, Morphogenesis, Transcription

## Abstract

The high mobility group-domain containing transcription factor Sox10 is an essential regulator of developmental processes and homeostasis in the neural crest, several neural crest-derived lineages and myelinating glia. Recent studies have also implicated Sox10 as an important factor in mammary stem and precursor cells. Here we employ a series of mouse mutants with constitutive and conditional Sox10 deficiencies to show that Sox10 has multiple functions in the developing mammary gland. While there is no indication for a requirement of Sox10 in the specification of the mammary placode or descending mammary bud, it is essential for both the prenatal hormone-independent as well as the pubertal hormone-dependent branching of the mammary epithelium and for proper alveologenesis during pregnancy. It furthermore acts in a dosage-dependent manner. Sox10 also plays a role during the involution process at the end of the lactation period. Whereas its effect on epithelial branching and alveologenesis are likely causally related to its function in mammary stem and precursor cells, this is not the case for its function during involution where Sox10 seems to work at least in part through regulation of the miR-424(322)/503 cluster.

## Introduction

Sox10 belongs to the Sox family of transcription factors. Its members are characterized by a sequence-specific DNA-binding high-mobility-group domain that was first identified in the mammalian male sex-determining factor Sry^1^. Like many other family members Sox10 is an important regulator of multiple developmental processes^2,3^. In particular, it has essential roles in the early neural crest and in several neural crest derivatives such as melanocytes, chromaffin cells, cells of the enteric nervous system and peripheral glia. Additionally, Sox10 regulates oligodendrocyte development in the central nervous system. Depending on cell type and time of development, it has been associated with maintenance of stem cell properties, survival of precursor cell populations, cell fate specifications and terminal differentiation processes^4–8^.

Recently, Sox10 has also been detected in the mammary gland^9^. Here it occurs in the mammary epithelium, a structure that arises from the ectoderm-derived mammary placode and invades into the mesenchymal mammary fat pad. In response to hormones, the mammary epithelium generates the ductal epithelial tree of the mammary gland during puberty by branching morphogenesis^10,11^. In the adult female, the bilayered ductal system undergoes further dramatic remodeling in response to hormones and local growth factor signaling^12^. The mammary epithelium strongly expands in pregnant mice, reaches its maximum during lactation and again decreases in size after weaning by apoptosis in a process termed involution^13^.

During prenatal mammary gland development, Sox10 is expressed in fetal mammary stem cells^9^. Sox10 persists in the adult gland where it is found in both basal and luminal layers of the mammary epithelium. Sox10 has been reported to occur in cells with progenitor properties at all stages of mammogenesis. Before birth, these are fetal mammary stem cells. In the adult, Sox10 is expressed in cells with features of bipotent mammary stem cells and unipotent luminal progenitor cells. In these cells, Sox10 is suggested to control stem cell properties in response to FGF signaling and to promote a mesenchymal-like state in a concentration-dependent manner^9,14^. SOX10 is also enriched in many triple-negative breast cancers and may thus represent a therapeutic target in this subgroup of breast cancers^9,15^.

Despite this recent characterization of Sox10 in mammary stem and progenitor cell populations, little is known about the biological role of Sox10 in the developing and adult mammary gland. Here, we use mice with constitutive or conditional *Sox10* null alleles to fill this gap in knowledge. As expected from its occurrence in stem cells and progenitor cells, we show an essential requirement of Sox10 for the expansion of the mammary epithelium. Additionally, our study revealed an unexpected function of Sox10 for the involution process, and we demonstrate that this function appears, at least in part, mediated by microRNAs from the miR-424(322)/503 cluster.

## Results

### Sox10 is required for branching of the mammary epithelium.

We have previously generated an allele in which the *Sox10* open reading frame is constitutively deleted and replaced by a *lacZ* reporter^4^. Mice carrying this allele have the advantage of exhibiting expression of the lacZ reporter in cells that normally express Sox10. In the homozygous state, *Sox10*^*lacZ/lacZ*^ mice die immediately after birth because of severe defects in the peripheral nervous system.

**Figure 1:**
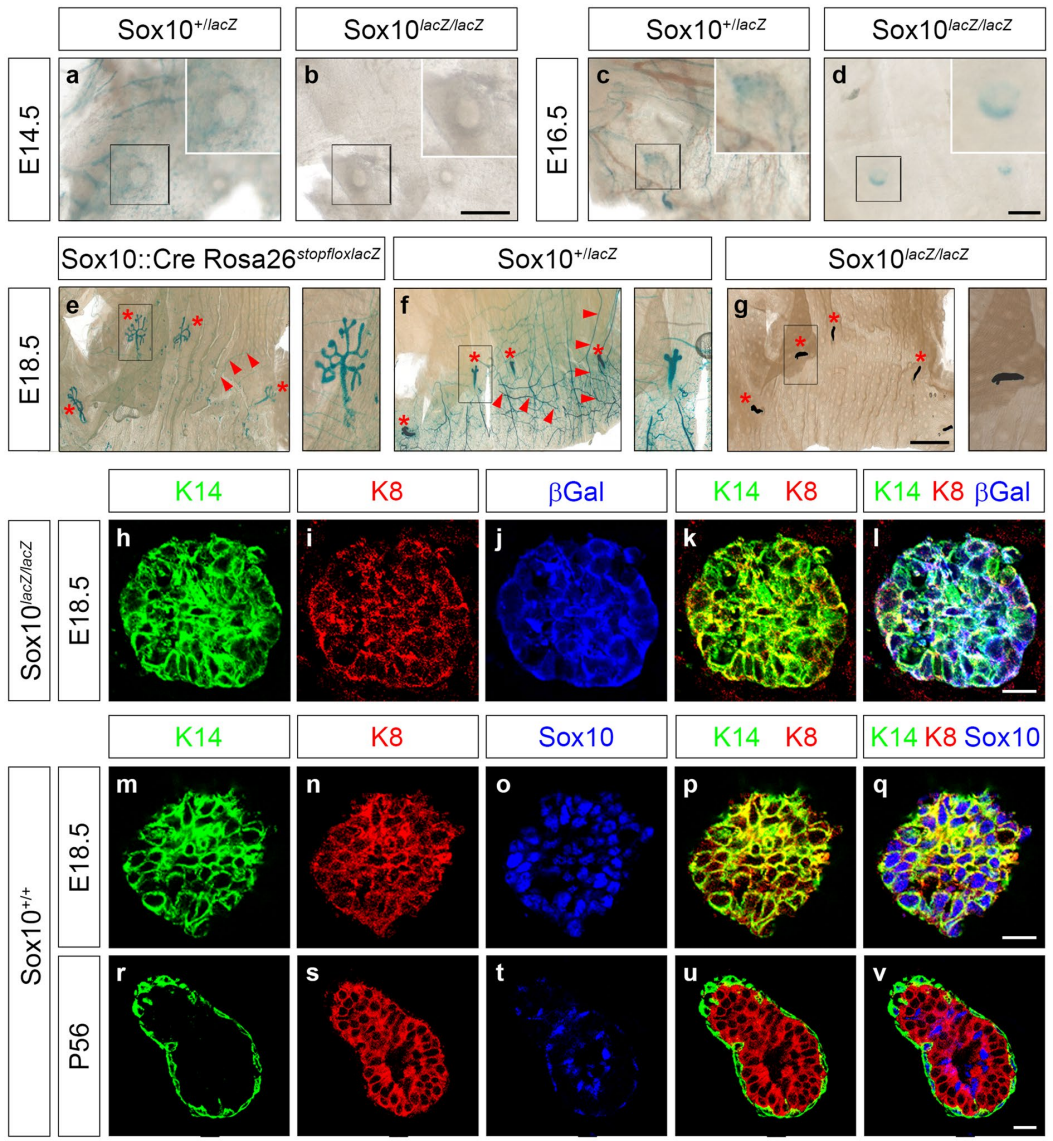
Prenatal mammary gland development in mice with embryonal Sox10 deletion. (**a**–**g**) X-gal staining was performed at E14.5 (**a**,**b**), E16.5 (**c**,**d**) and E18.5 (**e**–**g**) on ventral dermis including mammary gland anlagen to detect β-galactosidase activity in *Sox10*^+*/lacZ*^ (**a**,**c**,**f**), *Sox10*^*lacZ/lacZ*^ (**b**,**d**,**g**) and control *Sox10::Cre Rosa26*^*stopflox-lacZ*^ (**e**) mouse embryos. Staining is in the mammary gland anlagen (asterisks) and peripheral nerves (arrowheads). Magnifications in right upper corner (**a**–**d**) or on right (**e**–**g**) correspond to boxed areas in main panels. (**h**–**v**) Confocal sections of mammary gland ducts at E18.5 (**h**–**q**) and P56 (**r**–**v**) in Sox10^lacZ/lacZ^ (**h**–**l**) and *Sox10*^+*/*+^ (**m**–**v**) mice. Immunohistochemistry was performed using antibodies directed against keratin 14 (**h**,**m**,**r**), keratin 8 (**i**,**n**,**s**), β-galactosidase (βGal, **j**) and Sox10 (**o**,**t**). Single (**h**–**j**,**m**–**o**,**r**–**t**) or merged (**k**,**l**,**p**,**q**,**u**,**v**) stainings are shown. Scale bars: 50 µm (**b**,**d**), 2 mm (**g**), 10 µm (**l**,**q**,**v**).

To study the prenatal phase of mammary gland development in the absence of Sox10, we used *Sox10*^*lacZ/lacZ*^ embryos and performed X-gal staining of the ventral dermis in a region containing the anlagen of the mammary gland. For comparison, we stained *Sox10*^+*/lacZ*^ littermates. Additionally, we included age-matched *Sox10::Cre Rosa26*^*stopfloxlacZ*^ embryos in our analysis. These embryos also exhibit *lacZ* reporter expression in all Sox10-expressing tissues, but have two wildtype *Sox10* alleles. In *Sox10*^+*/lacZ*^ and *Sox10::Cre Rosa26*^*stopfloxlacZ*^ embryos we observed strong X-gal staining along all peripheral nerves because of Sox10 expression in Schwann cells (Fig. 1a,c,e,f). This peripheral nerve stain is missing in the *Sox10*^*lacZ/lacZ*^ embryos (Fig. 1b,d,g) as Schwann cells are not formed in the absence of Sox10^4^.

In all three genotypes, we counted 5 pairs of mammary buds in their normal position. At E14.5, the mammary anlagen in *Sox10*^*lacZ/lacZ*^ or *Sox10*^+*/lacZ*^ embryos looked grossly normal and did not exhibit X-gal staining (Fig. 1a,b). At E16.5, X-gal staining in the mammary bud region of *Sox10*^+*/lacZ*^ embryos still mostly originated from peripheral nerves and was barely above background in the structure itself (Fig. 1c). In comparison, buds of *Sox10*^*lacZ/lacZ*^ embryos were faintly but unambiguously stained (Fig. 1d). At E18.5, lacZ reporter activity was easily detectable within the buds in both genotypes (Fig. 1e–g). By this time, β-galactosidase staining was found in nearly all cells of the mammary epithelium where it was co-expressed with keratin 14 and keratin 8 (Fig. 1h–l). A comparable localization in all epithelial cells was found for Sox10 in wildtype embryos at E18.5 (Fig. 1m–q). At this time keratin 14 and keratin 8 were not yet restricted in their expression to basal or luminal layer of the mammary epithelium, as is the case at later times such as postnatal day (P) 56 when Sox10 expression is restricted to a subset of cells in either layer (Fig. 1r–v). These findings confirmed the exclusively epithelial expression of Sox10 in the developing mammary gland^9^. Considering the gross normal appearance of the early embryonic bud and the absence of substantial lacZ activity until late embryogenesis, Sox10 does not appear to be essential for mammary placode formation and early bud development. However, we cannot exclude more subtle structural alterations in the epithelial cell organization. It also remains formally possible that lacZ activity did not fully reflect Sox10 expression in the mammary bud for genetic or technical reasons. However, we like to point out that the *lacZ* allele has proven to be a very reliable indicator of Sox10 expression in many other cell types over the years^4,7,16^.

In *Sox10*^*lacZ/lacZ*^ embryos, epithelial sprouts invaded the mammary fat pad precursor. However, the sprouts in *Sox10*^*lacZ/lacZ*^ embryos failed to branch into the fat pad (Fig. 1g). Even in *Sox10*^+*/lacZ*^ littermates, branching was drastically impaired and the ductal tree consisted of much fewer branches at E18.5 than the one from *Sox10::Cre Rosa26*^*stopfloxlacZ*^ embryos (Fig. 1e,f). Thus, we conclude that Sox10 is required for proper epithelial branching during the prenatal period in a dose-dependent manner and for formation of a ductal tree.

**Figure 2:**
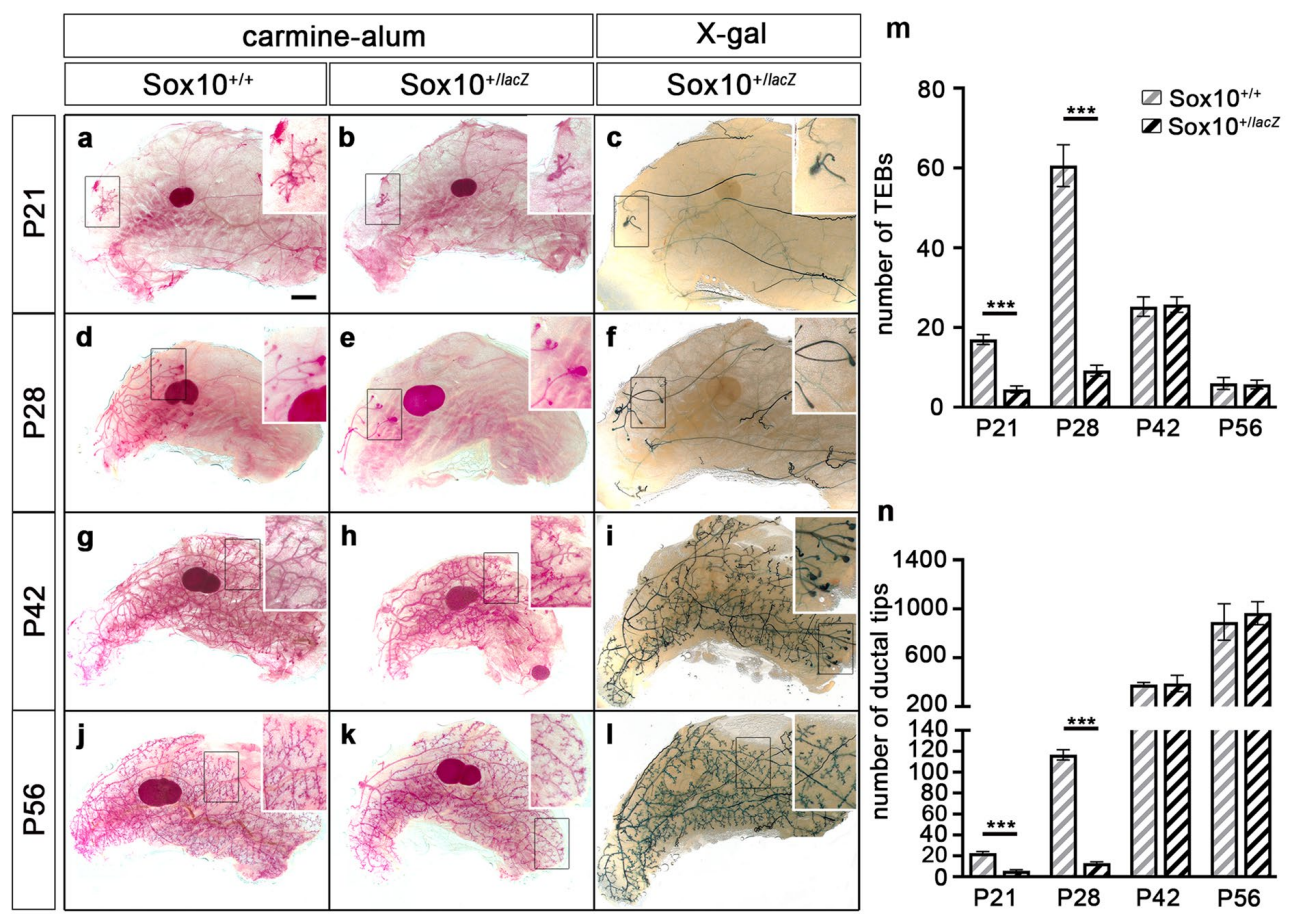
Postnatal mammary gland development in mice with embryonal Sox10 deletion. (**a**–**l**) Carmine-alum and X-gal stainings were performed at P21 (**a**–**c**), P28 (**d**–**f**), P42 (**g**–**i**) and P56 (**j**–**l**) on *Sox10*^+*/*+^ (wt in **a**,**d**,**g**,**j**) and *Sox10*^+*/lacZ*^ (**b**,**c**,**e**,**f**,**h**,**i**,**k**,**l**) mice. Inlays in right upper corner show magnifications of boxed areas in main panels. (**m**,**n**) The number of terminal end buds (TEB in m) and ductal tips (**n**) was quantified in mammary glands of *Sox10*^+*/*+^ (grey hatched bars) and *Sox10*^+*/lacZ*^ (black hatched bars) mice at P21, P28, P42 and P56 (n = 4–7). Presentations are as absolute cell numbers ± SEM. Statistical significance between age-matched *Sox10*^+*/*+^ and *Sox10*^+*/lacZ*^ was determined by Student`s t-test (***, P ≤ 0.001). Scale bar: 2 mm (**a**).

In the postnatal mammary gland, Sox10 function could only be studied in *Sox10*^+*/lacZ*^ mice. Until puberty, the mammary epithelium remained quiescent so that the ductal tree was still substantially less branched at postnatal day (P) 21 in female *Sox10*^+*/lacZ*^ mice than in wildtype mice as evident from carmine-alum and X-gal staining (Fig. 2a–c). Branching of the ductal epithelium into the fat pad was still impaired during the fourth postnatal week (Fig. 2d–f) with only 10 ± 1 terminal end buds and 13 ± 1 ductal tips per gland in *Sox10*^+*/lacZ*^ mice at P28 as compared to 61 ± 5 terminal end buds and 117 ± 5 ductal tips in the wildtype (Fig. 2m,n). However, by the end of the sixth week this difference between genotypes had disappeared (Fig. 2g–n) arguing that branching morphogenesis is delayed but not reduced in Sox10 heterozygous mice.

**Figure 3:**
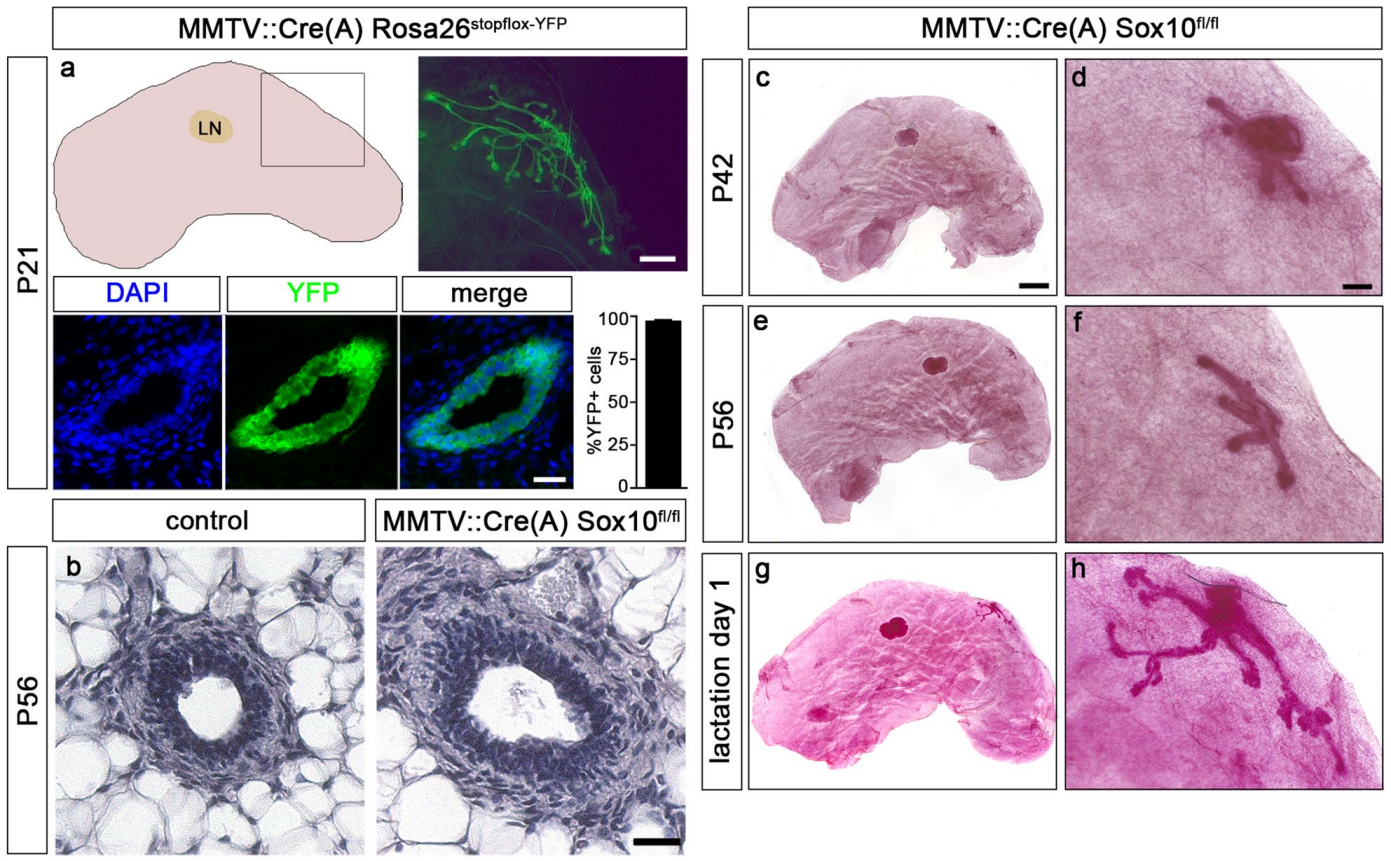
Mammary gland development in mice with early postnatal Sox10 deletion. (**a**) Mammary glands (schematically shown in the upper left panel including lymph node, LN) of *MMTV::Cre(A) Rosa26*^*stopfloxYFP*^ mice had undergone efficient Cre-dependent recombination at P21 as evident from YFP signals in whole mounts (upper right panel, corresponding to boxed area) and epithelial duct cross-sections (left lower panel) as well as quantification of YFP-positive cells ± SEM in the mammary epithelium (n = 3). DAPI was used to counterstain nuclei. (**b**) Ductal morphology in female control and *MMTV::Cre(A) Sox10*^*fl/fl*^ mice was analyzed by hematoxylin/eosin staining at P56. (**c**–**h**) Carmine-alum staining was used to visualize the mammary epithelium in female *MMTV::Cre(A) Sox10*^*fl/fl*^ mice at P42 (**c**,**d**) and P56 (**e**,**f**) or after 1 day of lactation (**g**,**h**). Panels on the left show the whole gland; panels on the right are magnifications of the area containing the mammary gland epithelium. Scale bars: 1 mm (**a**, upper panel), 100 µm (**a**, lower panel; **b**), 2 mm (**c**), 200 µm (**d**).

To further characterize the role of Sox10 in postnatal branching morphogenesis, we generated mice in which Sox10 was selectively deleted in the mammary epithelium by combining a floxed *Sox10* allele^6^ with a *MMTV::Cre* transgene^17^. We chose the *MMTV::Cre* transgenic line A over other lines because of its early expression in the postnatal mammary gland. Indeed, we confirmed that 97.7 ± 0.4% of epithelial cells in the mammary gland of *MMTV::Cre(A) Rosa26*^*stopfloxYFP*^ mice had undergone Cre-dependent recombination and activated expression of the YFP reporter at P21 (Fig. 3a). When we deleted Sox10, we first observed that *MMTV::Cre(A) Sox10*^*fl/fl*^ mice were severely underrepresented at the time of weaning. This was not unexpected as the *MMTV::Cre(A)* line is known to exhibit ectopic Cre expression in several tissues outside the mammary gland^18^. However, nine female *MMTV::Cre(A) Sox10*^*fl/fl*^ mice were obtained and analyzed in adolescence and early adulthood. At 6–8 weeks of age, these mice exhibited bilayered epithelial ducts with a lumen in their fat pads, but the ductal trees were rudimentary and had retained a size and a number of branches that resembled those of wildtype females at birth (Fig. 3b–f). Even after a full-term pregnancy, the ductal epithelium had hardly expanded (Fig. 3g,h). Dams were unable to lactate and feed their offspring. Considering the expression pattern of Sox10 and its known functions^19^, it is highly unlikely that a potential *MMTV::Cre(A)-*dependent *Sox10* deletion outside the mammary gland leads to systemic effects with impact on mammary gland development or physiology. The observed effects are much more likely due to Sox10 deletion in the mammary epithelium and suggest that Sox10 is essential for postnatal branching morphogenesis. A functional mammary gland does not form in the absence of Sox10.

### Reduced Sox10 levels impair mammary gland function.

**Figure 4:**
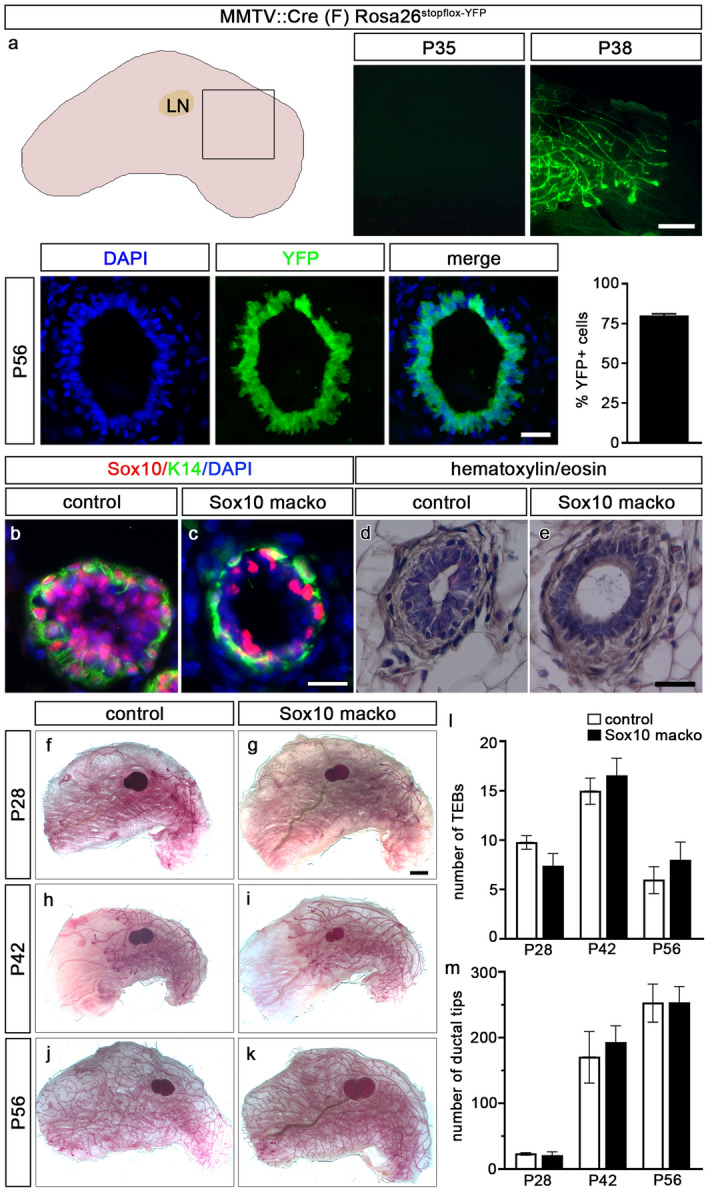
Mammary gland development in *Sox10 macko* mice with late adolescent Sox10 deletion. (**a**) Mammary glands (schematically shown on the upper left including lymph node, LN) of *MMTV::Cre(F) Rosa26*^*stopfloxYFP*^ mice underwent Cre-dependent recombination between P35 and P38 as evident from YFP signals (upper right panels, corresponding to boxed area). By P56, efficient Cre-dependent recombination had occurred as evident from YFP immunohistochemistry of epithelial duct cross-sections (left lower panel) as well as quantification of YFP-positive cells ± SEM in the mammary epithelium (n = 3). DAPI was used to counterstain nuclei. (**b**,**c**) Co-immunohistochemistry was performed on mammary ducts of control and *Sox10 macko* mice at 8 weeks of age using antibodies against Sox10 (red) and K14 (green). (**d**,**e**) Morphology of mammary ducts was compared in control and *Sox10 macko* mice at 8 weeks by hematoxylin/eosin staining. (**f**–**k**) Carmine-alum staining was performed at P28 (**f**,**g**), P42 (**h**,**i**) and P56 (**j**,**k**) on mammary glands of control (**f**,**h**,**j**) and *Sox10 macko* (**g**,**i**,**k**) mice. Scale bars: 2 mm (**a**, upper panel; **g**), 100 µm (**a**, lower panel), 50 µm (**c**,**e**). (**l**,**m**) The number of terminal end buds (TEB in l) and ductal tips (m) was quantified in mammary gland sections of control (white bars) and *Sox10 macko* (black bars) mice at P28, P42 and P56 (n = 4–7). Presentations are as absolute TEB and ductal tip numbers ± SEM. No statistical significance was observed between age-matched control and *Sox10 macko* mice by Student’s t-test.

To be able to analyze whether Sox10 is required after formation of the mammary gland in a virgin mouse, we switched to using the *MMTV::Cre* transgenic line F^17^. This transgenic line has been described to exhibit late Cre expression and activity in the mammary gland. This was indeed the case as Cre-dependent induction of a YFP reporter in *MMTV::Cre(F) Rosa26*^*stopfloxYFP*^ mice was virtually undetectable in the mammary gland at P35 (Fig. 4a). Only 3 days later, the YFP reporter had been efficiently induced. At P56, Cre-dependent deletion rates were 80.2 ± 1.0%. Although high, deletion rates were not as efficient as in *MMTV::Cre(A)* transgenic animals. Our results are in accord with the described incomplete penetrance of expression of the *MMTV::Cre(F)* transgene^17^. When interpreting data, the continued presence of a substantial number of non-recombined cells in the mammary epithelium has to be taken into consideration and poses a complication.

By combining the *MMTV::Cre(F)* transgene with floxed *Sox10* alleles, Sox10 could be deleted in the mammary gland of adult virgin mice. The resulting *MMTV::Cre(F) Sox10*^*fl/fl*^ mice were found after weaning in Mendelian ratios and are from now on referred to as *Sox10 macko* mice. Immunohistochemistry on the mammary ducts of *Sox10 macko* mice confirmed that Sox10-expressing cells were severely reduced, but not completely absent (compare Fig. 4b,c). The remaining Sox10-positive cells were found both in the keratin 14-positive basal layer and in the keratin 14-negative luminal layer of the ductal epithelium and were thus similarly arranged as the Sox10-positive cells in controls (Fig. 4b,c)^9^. Hematoxylin/eosin staining revealed no obvious abnormalities in the bilayered ductal structure in 8 week-old *Sox10 macko* mice (Fig. 4d,e).

In line with the time course of *Sox10* deletion, developing mammary glands exhibited no conspicuous abnormalities in *Sox10 macko* mice at four, six and eight weeks. Carmine-alum whole mount staining revealed that the fat pad was equally colonized by branching mammary epithelium in *Sox10 macko* and control mice both with regard to timing and pattern (Fig. 4f–k). The number of terminal end buds and ductal tips were comparable at all analyzed time points until week 8 (Fig. 4l,m).

**Figure 5:**
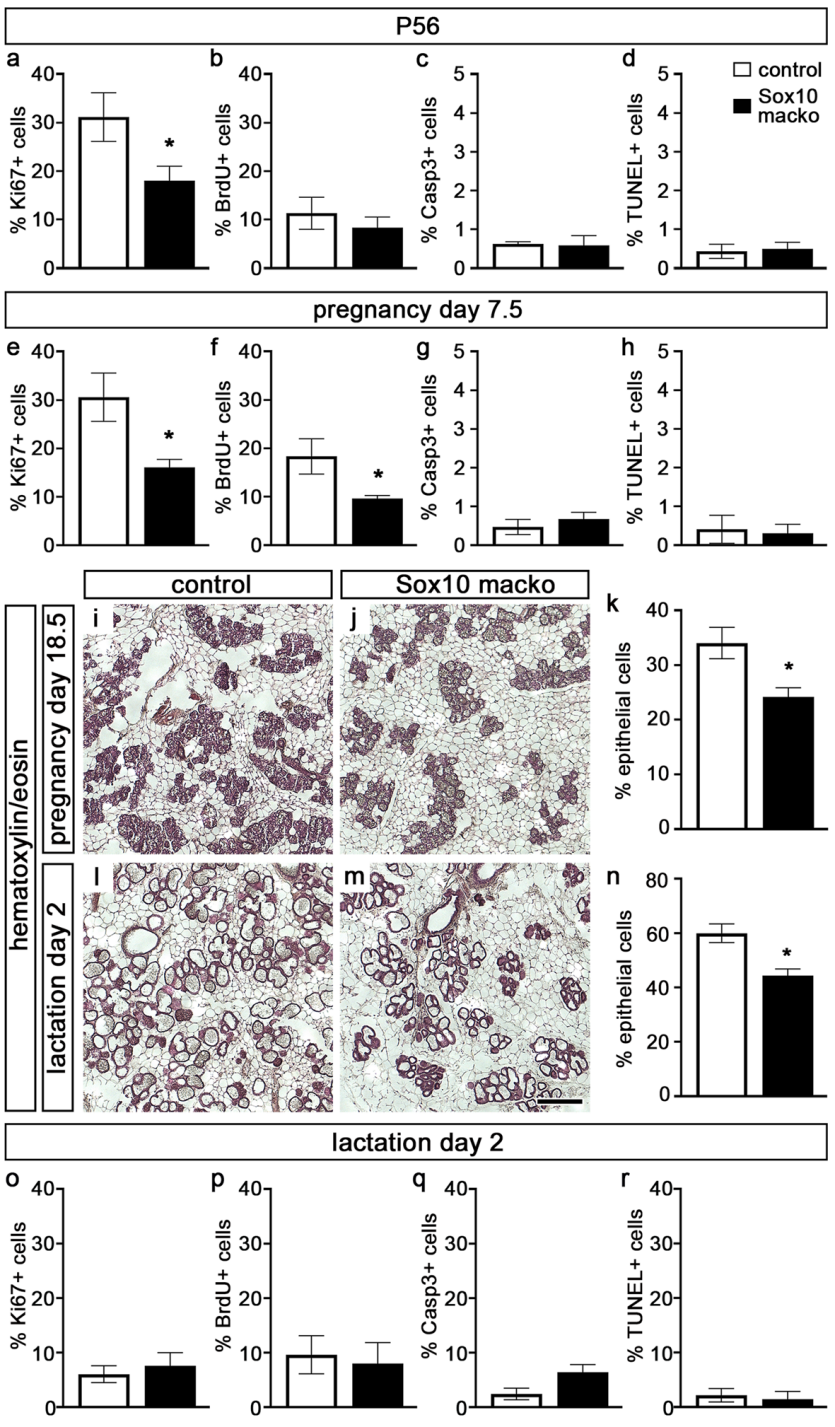
Proliferation and apoptosis in mammary glands of adult virgin, pregnant and early lactating *Sox10/tdTomato macko* mice. (**a**–**h**) Quantification of the percentage of tdTomato-positive mammary epithelial cells ± SEM in P56-old virgin (**a**–**d**) or 7.5 day pregnant adult (**e**–**h**) control (white bars) and *Sox10/tdTomato macko* (black bars) mice that are labelled by antibodies directed against Ki67 (**a**,**e**), BrdU (**b**,**f**), cleaved caspase 3 (**c**,**g**) or by TUNEL (**d**,**h**) (n = 5–10). (**i**–**n**) Hematoxylin/eosin staining (**i**,**j**,**l**,**m**) was performed on mammary gland sections to quantify the percentage ± SEM of epithelial cells in the gland (**k**,**n**) of 18.5 day pregnant (**i**–**k**) and 2 day lactating (**l**–**n**) adult control (white bars) and *Sox10 macko* (black bars) mice (n = 3–4). Scale bar: 500 µm (**j**). (**o**–**r**) Quantification of the percentage of mammary epithelial cells ± SEM in 2 day lactating control (white bars) and *Sox10/tdTomato macko* (black bars) mice that are labelled by antibodies directed against Ki67 (**o**), BrdU (**p**), cleaved caspase 3 (**q**) or by TUNEL (**r**) (n = 4–7). Statistical significance of differences between matched control and *Sox10 macko* was determined by Student`s t-test (*, P ≤ 0.05).

To investigate proliferation and cell death in the mammary epithelium of *Sox10 macko* mice, we crossed in an additional *Rosa26*^*stopfloxtdTomato*^ allele (*Sox10/tdTomato macko* mice), prepared single cell suspensions from mammary glands and quantified markers in tdTomato-positive recombined mammary epithelial cells. Mice with a combination of *MMTV::Cre(F)* transgene and *Rosa26*^*stopfloxtdTomato*^ allele served as controls. A lower percentage of the recombined mammary epithelial cells of adult virgin *Sox10/tdTomato macko* mice were Ki67-positive cells at eight weeks (Fig. 5a). There was also a trend towards lower BrdU incorporation in recombined mammary epithelial cells of 8-week-old *Sox10 macko* females relative to controls, although the difference did not reach statistical significance (Fig. 5b). Neither *Sox10/tdTomato macko* nor control mice had substantial numbers of mammary epithelial cells that stained positive for cleaved caspase 3 or TUNEL at eight weeks (Fig. 5c,d). We conclude from these findings that the proliferative capacity of Sox10-deficient epithelial cells in the mammary gland of adult virgin *Sox10 macko* mice was reduced. In contrast, apoptosis rates were normal. Direct counting of immunohistochemically stained mammary gland sections yielded comparable numbers.

During pregnancy, luminal cells expand substantially to give rise to secretory, milk producing alveoli. The percentage of Ki67-positive and BrdU-incorporating mammary epithelial cells of control mice remained high (Fig. 5e,f). Ki67-positive and BrdU-incorporating proliferative cells were significantly fewer among the recombined mammary epithelial cells of pregnant *Sox10/tdTomato macko* mice (Fig. 5e,f), whereas cell death was comparable to controls as determined by cleaved caspase 3 staining or TUNEL (Fig. 5g,h). As a consequence of the decreased rate of proliferation in the Sox10-deficient mammary epithelial cells of pregnant *Sox10 macko* mice, epithelial cells made up a smaller part of the gland shortly before and after giving birth than epithelial cells in control mice (Fig. 5i–n). At present, we do not know the contribution of Sox10-deficient recombined and Sox10-positive non-recombined mammary epithelial cells to the overall expansion of the mammary epithelium in pregnant *Sox10 macko* mice.

**Figure 6:**
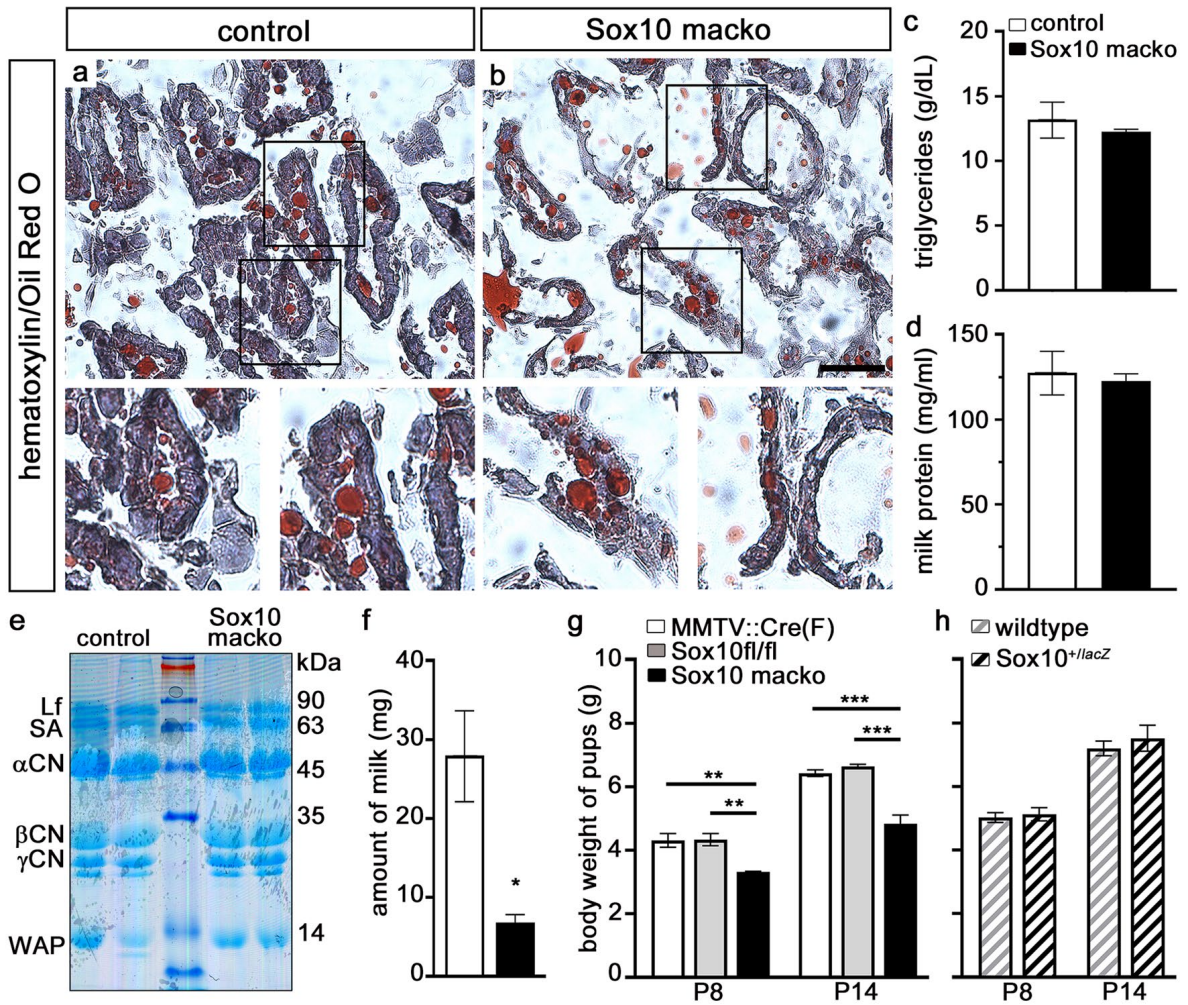
Analysis of mammary gland function in lactating *Sox10 macko* mice. (**a,b**) Oil Red O staining was performed in combination with hematoxylin/eosin staining on mammary gland sections of 14 day lactating adult control (**a**) and *Sox10 macko* (**b**) mice. Magnifications at bottom correspond to boxed areas in main panels. (**c**) Triglyceride content of milk from lactating adult control (white bars) and *Sox10 macko* (black bars) mice was determined as g/dl ± SEM (n = 3–4). (**d**) Protein content of milk from lactating adult control (white bars) and *Sox10 macko* (black bars) mice was measured in mg/ml ± SEM (n = 3–4). (**e**) Composition of milk proteins in lactating adult control and *Sox10 macko* mice was analyzed by SDS polyacrylamide gelelectrophoresis and Coomassie blue staining. Sizes of molecular weight markers are indicated on the right in kilodaltons (kDa). CN, casein, Lf, lactoferrin; SA, serum albumin; WAP, whey acid protein. (**f**) The amount of milk retrievable from lactating adult control (white bars) and *Sox10 macko* (black bars) mice is presented in mg per milking event (4 animals per genotype, 3 milking events per animal). (**g,h**) The body weight of pups from *MMTV::Cre(F)* mothers (white bars, **g**), *Sox10*^*fl/fl*^ mothers (grey bars, **g**), *Sox10 macko* (black bars, **g**), *Sox10*^+*/*+^ (grey hatched bars, **h**) and *Sox10*^+*/lacZ*^ (black hatched bars, **h**) mothers was determined at P8 and P14 and is presented in g ± SEM per pup (4–9 size-matched litters per genotype with 4–8 pups per litter.) Scale bar, 100 µm. Statistical significance of differences among genotypes was determined by Student`s t-test (**c,d,f,h**) or One-way ANOVA with Bonferroni’s multiple comparisons test (**g**) (*, P ≤ 0.05; **, P ≤ 0.01; ***, P ≤ 0.001).

Early into the lactation period, rates of proliferation and apoptosis were comparable between *Sox10/tdTomato macko* and control mice (Fig. 5o–r). The presence of lipid-rich droplets in alveolar cells and the lumen of alveoli was assessed using Oil Red O staining (Fig. 6a,b). Visual inspection of mammary gland sections indicated that the distribution of the lipid-rich material was altered in *Sox10 macko* mice. We observed more lipids within alveolar cells as compared to controls (6.2 ± 1.2 droplets in epithelial cells per alveolus in *Sox10 macko* compared to 0.6 ± 0.2 in controls counting 20–27 alveoli per animal with 3 animals per genotype), suggesting that *Sox10 macko* females may have a defect in secretion.

Lactating *Sox10 macko* mice were able to produce milk. Our analyses of its chemical composition revealed that the content of triglycerides in the milk was similar between *Sox10 macko* and wildtype females (Fig. 6c). There was also no difference in the overall protein mass (Fig. 6d). Moreover, gel electrophoresis of milk proteins demonstrated that all major protein fractions were present in milk from *Sox10 macko* mice including lactoferrin, serum albumin, whey acid protein, and caseins (Fig. 6e; for full gel see also Supplementary Fig. 1). While no obvious changes in the relative amounts of milk proteins were detected, lactating *Sox10 macko* mice produced statistically significant smaller amounts of milk (Fig. 6f). As a consequence, pups in size-matched litters with at least 4 pups gained less weight during the first two weeks when they were nursed by *Sox10 macko* dams compared to pups nursed by wildtype females or dams that carried only the *MMTV::Cre(F)* transgene (Fig. 6g). The difference in weight gain was independent of the genotype of the pups. A reduction in weight gain was not observed for pups nursed by Sox10^+/lacZ^ dams (Fig. 6h). These findings suggest that the reduced proliferation of mammary epithelial cells in the absence of Sox10 led to an impaired expansion of the mammary epithelium during pregnancy and reduced milk production during lactation in *Sox10 macko* mice. Our results also indicate that reduction to one functional *Sox10* allele per epithelial cell is not sufficient to obtain this effect.

### Sox10 is required for involution of the mammary epithelium.

**Figure 7:**
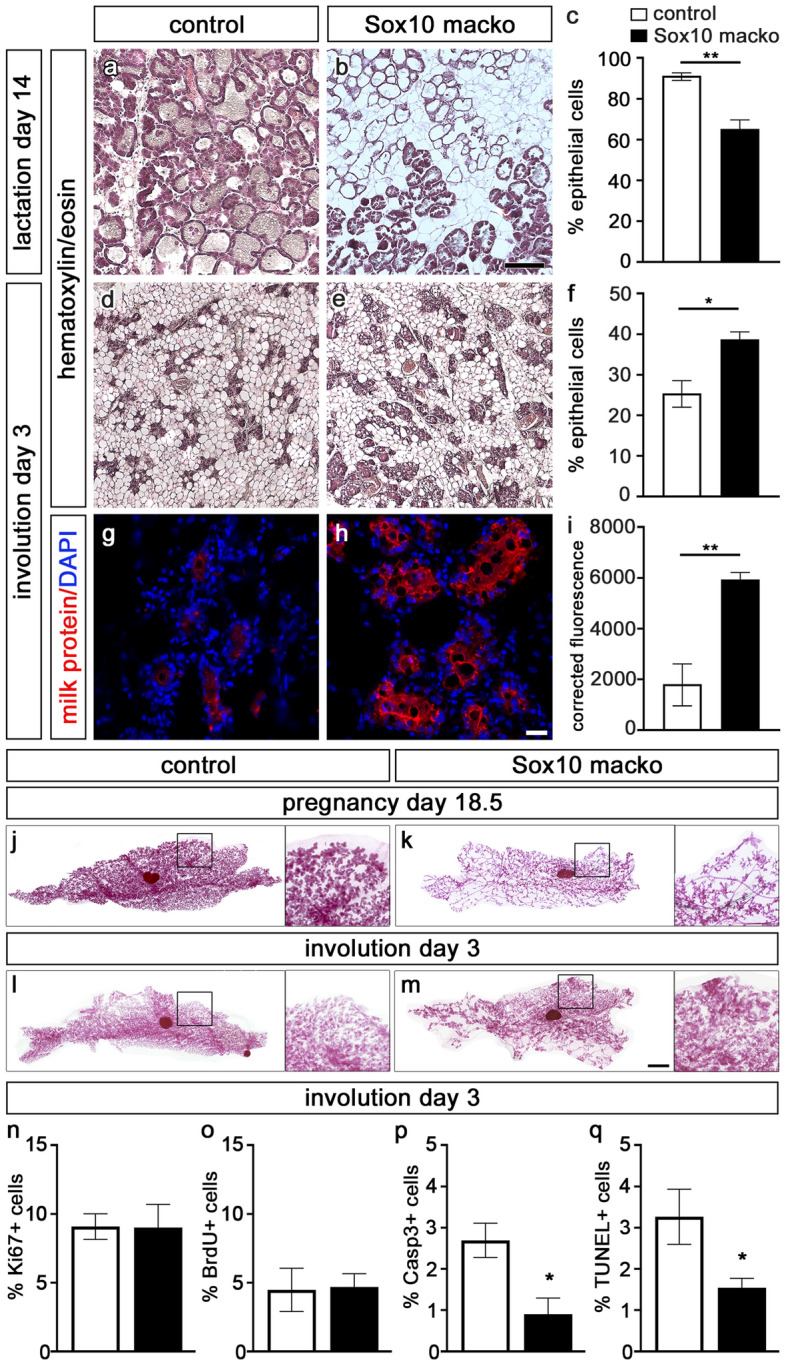
Analysis of involution of the mammary gland in *Sox10 macko* mice. (**a**–**f**) Hematoxylin/eosin staining of mammary gland sections was performed to compare the histology in adult control (**a**,**d**) and *Sox10 macko* (**b**,**e**) mice at 14 days of lactation (**a**–**c**) and 3 days of involution (**d**–**f**) and to quantify the percentage ± SEM of epithelial cells in the gland (**c**,**f**) (n = 3–5). (**g**–**i**) Immunohistochemistry with antibodies (red) directed against mouse milk proteins (**g**,**h**) was used to quantify the milk protein signal on mammary gland sections of adult control (white bars) and *Sox10 macko* (black bars) mice at 3 days of involution as corrected fluorescence ± SEM. (**i**). Nuclei were counterstained with DAPI. (n = 3–4). (**j**–**m**) Carmine-alum stainings were performed at day 18.5 of pregnancy (**j**,**k**) and day 3 of involution (**l**,**m**) on control (**j**,**l**) and *Sox10 macko* (**k**,**m**) mice. Magnifications on right correspond to boxed areas in main panels. Scale bars: 500 µm (**b**), 50 µm (**h**), 1 mm (**m**). (**n**–**q**) Quantification of the percentage of mammary epithelial cells ± SEM at day 3 of involution in control (white bars) and *Sox10/tdTomato macko* (black bars) mice that are labelled by antibodies directed against Ki67 (**n**), BrdU (**o**), cleaved caspase 3 (**p**) or by TUNEL (**q**) (n = 5–8). Statistical significance of differences between control and *Sox10 macko* was determined by Student’s t-test (*, P ≤ 0.05; **, P ≤ 0.01).

Following the weaning of the offspring, the mammary epithelium involutes and undergoes a dramatic remodeling process. Whereas epithelial cells made up 91 ± 2% of all cells in the mammary gland of control mice at the peak of lactation (Fig. 7a,c), they were reduced to a mere 25 ± 3% at the third day of involution (Fig. 7d,f). Intriguingly, these changes were much less pronounced in *Sox10 macko* mice. In these mutant females, the contribution of epithelial cells declined from 65 ± 4% during lactation (Fig. 7b,c) to 40 ± 2% at day 3 of involution (Fig. 7e,f). These differences in remodeling were also visible in whole mount carmine alum stainings of mammary glands from control and *Sox10 macko* mice (Fig. 7j–m). Although the mammary gland in *Sox10 macko* mice had a lower percentage of secretory epithelial cells compared to mammary glands of controls at the day of birth and during lactation, the epithelial cells persisted longer in *Sox10 macko* mice during involution. This surprising observation suggested that Sox10 may play an unanticipated biologically relevant role in the involution process.

To study this phenomenon in more detail, we performed an immunohistochemical staining of milk proteins on histological sections of involuting mammary glands, and we observed that the mammary glands of *Sox10 macko* females retained a significantly elevated expression of milk proteins at day 3 of involution. In contrast, the mammary glands of controls contained only low amounts of milk proteins at this stage of the remodeling process (Fig. 7g–i). Using immunocytochemical analysis of single cell preparations of mammary glands from *Sox10/tdTomato* macko and control mice at day 3 of involution, we determined proliferation and cell death rates (Fig. 7n–q). Whereas proliferation rates in mammary epithelial cells were comparably low between genotypes, rates of dying cells were significantly lower in the mammary epithelium of *Sox10/tdTomato macko* mice compared to controls as determined by cleaved caspase 3 staining and TUNEL (Fig. 7n–q). Therefore, the delayed involution in *Sox10 macko* mice is caused primarily by changes in programmed cell death of the secretory epithelium.

**Figure 8:**
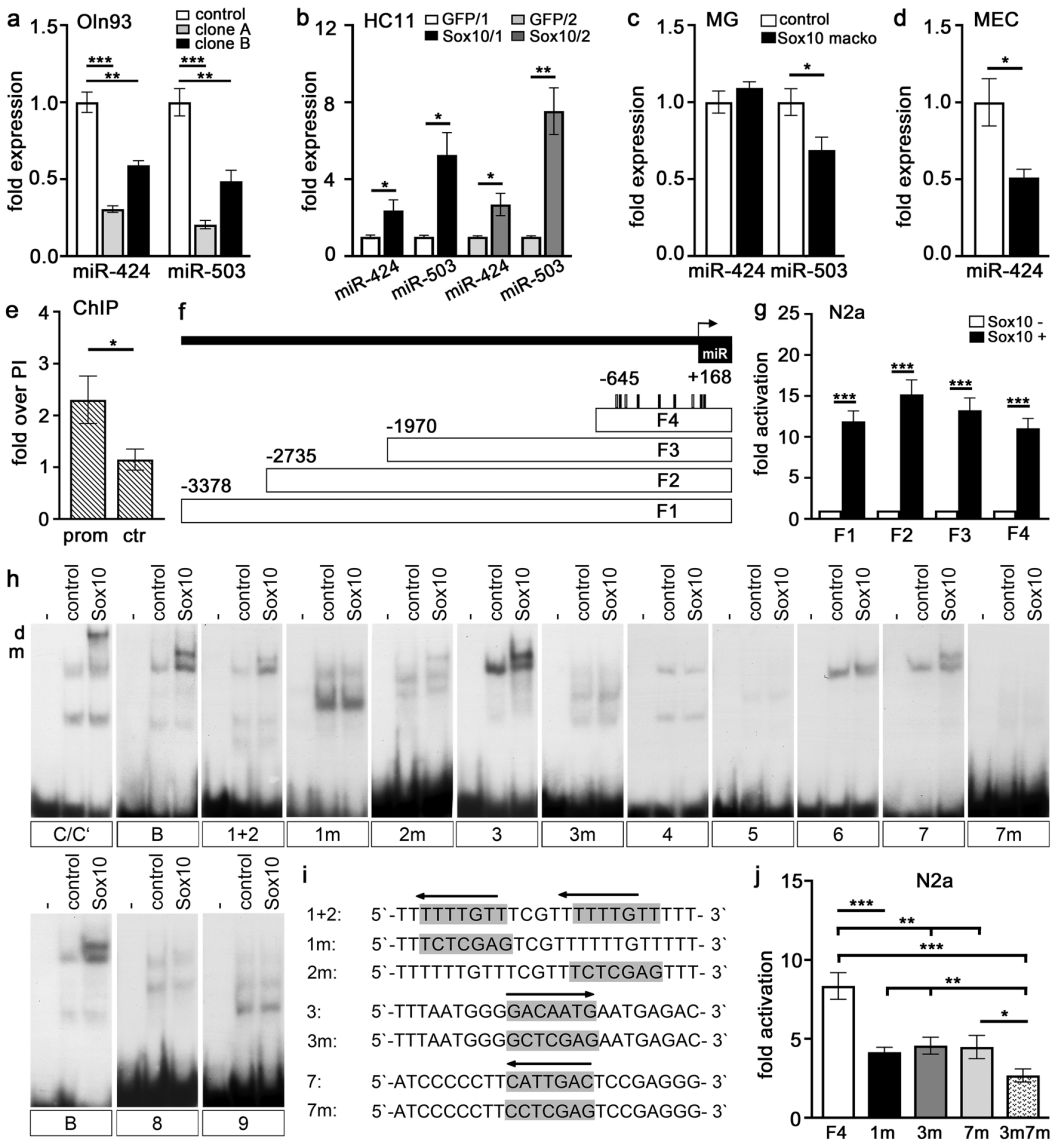
The miR-424(322)/503 cluster is a direct downstream target of Sox10. (**a**–**d**) Relative expression levels ± SEM of miR-424 and miR-503 were determined in control (white bars) and Sox10-deficient (grey and black bars) Oln93 cells (**a**), control (white and light grey bars) and Sox10-overexpressing (dark grey and black bars) HC11 cells (**b**), involuting control (white bars) and *Sox10 macko* (black bars) mammary glands (MG, **c**) and FACS-purified mammary epithelial cells (MEC, **d**) from control (white bars) and *Sox10/tdTomato macko* (black bars) mice (n = 3–4). (**e**) Chromatin immunoprecipitation on involuting mammary glands with antibodies against Sox10. Fold enrichment ± SEM of the miR-424(322)/503 promoter (prom) and a genomic control region in the immunoprecipitates was determined relative to preimmune serum (PI; n = 3). (**f**) Scheme of the miR-424(322)/503 promoter (F1, positions − 3378 to + 168) and its subfragments F2–F4. Positions of potential Sox10 binding sites in F4 and transcriptional start site (arrow) are indicated. (**g**) Fold activations ± SEM were determined for luciferase reporter plasmids carrying miR-424(322)/503 promoter fragments in transiently transfected N2a in the presence or absence of Sox10 (n ≥ 6). (**h**) For EMSA experiments, oligonucleotides containing potential Sox10 binding sites 1–9 (each on a separate gel; for localization, see (**f**) were incubated without cell extract (−), or in the presence of extracts from HEK293 cells transfected with empty (control) or Sox10 expression vector. Binding sites C/C′ and B from the *Mpz* gene served as control for dimeric and monomeric binding. Sox10 binding to sites 1, 2, 3 and 7 was further analyzed by introducing mutations. (**i**) Sequences of Sox10 binding sites 1, 2, 3, 7 and their mutant versions 1m, 2m, 3m and 7m. (**j**) Fold activations ± SEM were determined for luciferase reporter plasmids carrying the F4 core promoter fragment in wildtype version (F4) or after mutation of sites 1, 3 and 7 (1m, 3m, 7m, 3m7m) in transiently transfected N2a in the presence or absence of Sox10 (n ≥ 8). Statistical significance was determined by Student’s t-test (**a**–**e**,**g**) or One-way ANOVA with Bonferroni’s multiple comparisons test (**j**) (*, P ≤ 0.05; **, P ≤ 0.01; ***, P ≤ 0.001).

The miR-424(322)/503 cluster has been reported to orchestrate epithelial remodeling in the involuting mammary gland^20^. In a screen for microRNAs with Sox10-dependent expression, we had previously found that microRNAs of the miR-424(322)/503 cluster were moderately downregulated in the absence of Sox10 in the oligodendroglial cell line Oln93^21^. RT-PCR experiments confirmed that miR-424 and miR-503 were both expressed at higher levels in wildtype Oln93 cells than in Oln93 clones that had undergone CRISPR/Cas9-dependent Sox10 inactivation (Fig. 8a). To investigate whether comparable changes in microRNA expression may occur in mammary epithelial cells, we also analyzed the expression of the miR-424(322)/503 cluster in HC11 cells^22^. As HC11 cells express only low levels of Sox10, we used retroviral transduction followed by FACS to generate Sox10-positive HC11 cells. HC11 cells were then cultivated and lactogenic differentiation was induced before RNA-preparation. Consecutive RT-PCR experiments showed that Sox10 was able to robustly stimulate the expression of miR-424 and miR-503 in HC11 cells (Fig. 8b). When RT-PCR analyses were performed on RNA from whole involuting mammary glands of *Sox10 macko* and control mice, statistically significant differences were detected for miR-503, but not for miR-424 expression levels (Fig. 8c). We attribute our failure to detect differences in miR-424 expression to the fact that the presence of this particular miRNA is not confined to the mammary epithelium. In line with this assumption, differences in expression levels became detectable for miR-424 when mammary epithelial cells were isolated by FACS from *Sox10/tdTomato macko* mice and *Rosa26*^*stopfloxtdTomato*^* MMTV::Cre(F)* controls and used as source of RNA (Fig. 8d). Levels of miR-503 were reduced in mammary epithelial cells of *Sox10/tdTomato macko* mice to 59.1 ± 26.5% of controls.

As these experiments indicated that the miR-424(322)/503 cluster may be a direct transcriptional target of Sox10, we focused on the previously identified promoter region of this cluster^20^. Chromatin immunoprecipitation experiments with antibodies directed against Sox10 and chromatin prepared from involuting mammary glands showed selective enrichment of this promoter region in the precipitate (Fig. 8e). The same promoter was also robustly stimulated by Sox10 in luciferase reporter assays after transient transfection (Fig. 8f,g). Successive shortening of the 3.5 kb promoter fragment furthermore revealed that the 0.8 kb core promoter was still activated more than tenfold by Sox10 (Fig. 8f,g). This region contained nine nucleotide sequences with less than two mismatches to the Sox consensus sequence outside the essential CAA core of the binding motif (Fig. 8f). We performed EMSA to determine whether these sequences are binding sites for Sox10. The results showed that only sites 1, 3 and 7 exhibited a robust binding of Sox10 (Fig. 8h). Mutational changes of these sites abolished their interaction with Sox10 and confirmed the exact location of the Sox10 binding sites (Fig. 8h,i). Introduction of the same mutations into single sites in the context of the core promoter of the miR-424(322)/503 cluster reduced Sox10 responsiveness by approximately 50% (Fig. 8j). A combination of mutations within sites 3 and 7 abolished Sox10 responsiveness even further, suggesting that a direct binding of Sox10 to several sites is responsible for activation of the promoter of the miRNA cluster. The collective results from this line of investigation strongly suggest but do not unequivocally prove that Sox10 regulates the expression of miR-424 and miR-503 as a part of the mechanism by which Sox10 exerts its biological effect on the involution process.

## Discussion

Previous work had shown that Sox10 is expressed in stem and precursor cell populations of the mammary epithelium where it promotes stem cell properties and epithelial-mesenchymal transition in a concentration- and context-dependent manner^9,14^. In support of this notion, Sox10 had also been found to be enriched in basal-like and EMT-like subtypes of triple-negative breast tumors. Despite these important scientific insights, the potential roles of Sox10 in normal mammary gland development had not been investigated.

In this study, we show that Sox10 is an important transcription factor that controls the development and cellular homeostasis of the mammary gland. When studying mice with a constitutive *Sox10* deletion, we found no evidence for a role of Sox10 in the specification of the mammary line or the formation of mammary placodes, epithelial buds and epithelial sprouts. This may in part be due to low Sox10 expression in mammary placode and early bud. Alternatively, loss of Sox10 may be compensated by its close relative Sox9 that has also been reported to be expressed in the early mammary anlagen^23^.

Similarly to Lrp6 and Pygo2 knockout mice^24,25^, the epithelial sprouts of Sox10 conditional knockouts exhibited an early arrest in branching and growth, suggesting that Sox10 is required for mammogenesis during late embryonic development. The similarity of the phenotypic defects with Lrp6 and Pygo2 knockout mice suggests a link to canonical Wnt signalling, which remains to be investigated in future studies.

Interestingly, branching was also strongly reduced in mice with a heterozygous Sox10 deficiency. This argues for a dose-dependent Sox10 function. A similar dose-dependency has been previously detected for other Sox10 functions in several neural crest derivatives and becomes evident as haploinsufficiency in mice and men^26–29^.

In mice with floxed Sox10 alleles, the initial branching of the mammary epithelium occurred normally. However, the deletion of *Sox10* in juvenile mice using the *MMTV::Cre(A)* line resulted in a failure of the mammary epithelium to further expand the initial ductal tree during puberty. This clearly suggests that the role of Sox10 in epithelial branching morphogenesis is not restricted to the prenatal phase, but equally relevant to the second phase of expansion during puberty. It remains to be studied how Sox10 interacts with the pathways that control this expansion including the estrogen, progesterone, growth hormone and EGF receptor pathways^13^. It is reasonable to assume that the previously established role of Sox10 in stem cell maintenance may be relevant in the branching process^9^.

The severity of the phenotype caused by the early postnatal deletion of Sox10 did not allow the investigation of Sox10 function during later stages of mammogenesis. We therefore also deleted this transcription factor from the mammary epithelium in the fully developed mammary gland of virgin mice using the *MMTV::Cre(F)* transgene. Despite residual expression of *Sox10* in some epithelial cells, we observed phenotypic abnormalities in alveologenesis during pregnancy. Consequently, Sox10-deficient mammary glands contained fewer alveoli in lactating mice and produced less milk. The protein and lipid composition of the milk, however, remained unchanged. The analysis of various markers indicated that apoptosis rates were normal in Sox10-deficient mammary glands of virgin mice. However, these glands contained fewer proliferative epithelial cells, and were unable to enlarge the lobuloalveolar compartment to wildtype levels during pregnancy.

Interestingly, we did not only observe an impaired expansion of the mammary epithelium during pregnancy, but also a defect in the involution process following the weaning of the offspring. This was unexpected and cannot easily be explained by the proposed role of Sox10 in mammary stem/precursor cells. It argues that Sox10 may have an additional, so far unknown role in the involution process despite the fact that it is expressed at substantially lower levels than during pregnancy and lactation^30^. This is not uncommon for Sox10 as multiple stage-specific roles have been observed for this transcription factor in other cell types such as Schwann cells where expression also varies between stages^4,31,32^. Involution is regulated by the microRNAs encoded in the miR-424(322)/503 cluster^20^. Since our previous studies showed that Sox10 regulated the expression of this miRNA cluster in neural cells, we investigated whether this regulatory relationship would also apply to mammary epithelial cells. The analysis of Sox10-deficient mammary glands and a mammary epithelial cell line with differential expression of Sox10 supports this notion. Moreover, our molecular studies pointed to the promoter of the miR-424(322)/503 cluster as a Sox10-responsive regulatory region, and the presence of 3 functional binding sites for Sox10 argued for a direct effector-target gene relationship between Sox10 and the miR-424(322)/503 cluster. We note that Sox10 expression peaks during pregnancy and lactation, whereas expression of miR-424 and miR-503 is highest during involution^20,30^. In the absence of a good correlation between effector and target gene expression, we postulate that the interaction with temporally and spatially restricted transcription factors is a major determinant of Sox10 function during involution. The relevance of such interactions for stage-specific Sox10 functions has been documented in other cell types such as oligodendroglia^33,34^. Previous studies had shown that miR-424 and miR-503 function downstream of TGF-β during involution^20^. Therefore, it will be interesting to see whether Sox10 is involved in mediating the TGF-β effects or whether it works in a parallel pathway.

Similar to Sox10, the closely related Sox9 has also been implicated in mammary gland development, maintenance of the mammary stem cell state and breast cancer^23,35,36^. Co-expression of the two closely related, structurally highly homologous Sox proteins is not uncommon^19^. In cases of co-expression, both proteins have been shown to either function similarly or antagonistically^37,38^. In the mammary gland, both Sox9 and Sox10 are expressed in stem/precursor cells of the epithelial compartment and appear to have substantially overlapping functions. Therefore, we would have expected them to be largely redundant. However, the strong developmental phenotypes in the single knockouts^23^^(this study)^ argue against this idea, at least beyond the early embryonic stage.

To explain the requirement for both Sox9 and Sox10 we entertain two alternative scenarios. Considering that Sox9 has been reported to induce *Sox10* expression in mammary epithelial cells, one possible explanation is that Sox9 acts upstream of Sox10^35^. Given that Sox9 has been primarily localized in luminal precursors, whereas Sox10 has been found in both basal and luminal precursor cells, another possibility is that Sox10 is more important in basal and Sox9 more important in luminal precursor cell populations. If Sox10 and Sox9 have discrete functions within basal or luminal progenitors in the normal gland, they may also have discrete functions in breast cancer subtypes that differ in their contributions of basal and luminal cells. This needs to be clarified in future experiments.

## Methods

### Generation of mice, genotyping and husbandry

Mice either carried the *Sox10*^*lacZ*^ allele as a constitutive Sox10 knockout allele (MGI Cat# 2151173, RRID:MGI: 2151173)^4^ or *Sox10*^*fl*^ (MGI Cat# 4820417, RRID:MGI: 4820417), *Rosa26*^*stopfloxlacZ*^ (MGI Cat# 1861931, RRID:MGI: 1861931), *Rosa26*^*stopfloxYFP*^ (MGI Cat# 2449038, RRID:MGI: 2449038) and *Rosa26*^*stopfloxtdTomato*^ (MGI Cat# 3809524, RRID:MGI: 3809524) as floxed alleles^6,39–41^. One copy of the *Rosa26* reporter alleles or two copies of the *Sox10*^*fl*^ allele were combined with *Sox10::Cre* (MGI Cat# 3586900, RRID:MGI: 3586900) or *MMTV::Cre lines* A and F (MGI Cat# 3802643, RRID:MGI: 3802643) in analyzed mouse mutants^17,42^. Genotyping was performed as described^6,17,39,41^. All mice were on a mixed C3H × 129/SvJ background. They were kept under standard housing conditions with 12:12 h light–dark cycles and continuous access to food and water in accordance with animal welfare laws.

Only females were used for the study. In the period between weaning and fertilization, females were kept in a separate room with transgenic animals and controls in the same cage to synchronize their estrous cycle. For timed pregnancies adult female mice were mated. The morning when a vaginal plug was detected, was defined as day 0.5 for the embryo and the pregnant mother. Ventral dermis including the mammary anlagen used for X-gal stainings were prepared from E14.5, E16.5 and E18.5 embryos. For analysis of the mammary anlagen, embryos were killed, divided in half and fixed for 1.5 h in 4% paraformaldehyde. Inner organs were removed and fixation was continued in 1% paraformaldehyde overnight. Fore limbs and hind limbs were removed before X-gal staining^4^.

For growth analysis of pups litters were size-matched among genotypes and consisted of 4–8 pups per litter. Weight gain was recorded during the first two postnatal weeks. For analysis of amounts and composition milk was collected as described^43^. Briefly, nursing dams were separated from their pups by isolating pups in small confinements inside their dam`s cage for four hours. Afterwards physical contact between dams and pups was allowed for several minutes to stimulate release of oxytocin. Dams were then anesthetized, and milk ejection was stimulated by gentle finger massage. From each dam milk was obtained from 3 time points between the first and second week of lactation.

Mammary glands were prepared from mice during puberty, adolescence and in the adult in the virgin, pregnant, lactating or involution stage. For involution studies, pups were removed at day 5 of lactation and dams killed 3 days later. Mammary glands were fixed overnight in 1% paraformaldehyde when used for X-gal staining or in 4% paraformaldehyde when used for immunohistochemistry or histological staining. For quantifications and FACS, mammary glands were immediately dissociated and incubated for 2-3 h in Dulbecco’s Modified Eagle’s Medium (DMEM) F12 containing 10% FCS, 1.5 mg/ml collagenase type 2 (Worthington, CLS-2, Lot F8K18521) and 125 U/ml hyaluronidase (Sigma #3606, Lot SLB1921). Lymph nodes in abdominal glands were removed before treatment. Dissociated glands were pelleted, washed with PBS and resuspended in red blood cell lysis buffer (Invitrogen/eBioscience, Lot 2094600). After centrifugation and washing with PBS, cells were resuspended in DMEM F12 containing 10% FCS in the presence of penicillin and streptomycin and either used for FACS or seeded at a density of 70,000 cells per cm^2^ onto poly-D-lysine-coated glass slides. After overnight incubation at 37 °C and 5% CO_2_, adherent cells were quantified. BrdU was added at 10 µM final concentration 4 h before fixation.

### Immunohistochemistry, immunocytochemistry and BrdU incorporation assays

For immunohistochemical analysis paraformaldehyde-fixed mammary glands (n = 3–7 per condition) were washed with PBS, embedded in OCT compound and sectioned at 10 µm on a Leica CM1950 cryotome. Antigen-retrieval (except for anti-milk protein staining) was performed by boiling in Tris/EDTA pH 9.0 for 15 min. Sections were blocked in 10% fetal calf serum (FCS) and 1% bovine serum in PBS for 2 h and incubated with primary antibodies overnight at 4 °C. The following primary antibodies were applied: guinea pig anti-Sox10 antiserum (1:1,000 dilution^44^, RRID: AB_2721917), rabbit anti cleaved caspase 3 antiserum (1:200 dilution, Cell Signaling #9661, RRID: AB_2341188), rabbit anti-keratin 14 antiserum (1:500 dilution, BIOZOL Diagnostica PRB-155P, RRID: AB_292096), rabbit anti-keratin 8 antiserum (1:200 dilution, Abcam ab194130, RRID: AB_2728659), rabbit anti-Ki67 antibodies (1:1000 dilution, LabVision/Neomarkers RM-9106, RRID: AB_2335745), rabbit anti-mouse milk protein antiserum (1:500, Accurate Chemical YNRMMSP, no RRID available). Secondary antibodies were coupled to Cy3, Cy5 (Dianova) or Alexa488 (Millipore) fluorescent dyes. For BrdU labeling, adult virgin mice were injected with 100 μg of BrdU (Sigma) per gram body weight 24 h before preparation, whereas pregnant mice were injected 6 h before preparation. Incorporated BrdU was detected on mammary gland sections using a rat monoclonal antibody directed against BrdU (Abcam ab6326, RRID:AB_305426) at a 1:20 dilution. The secondary antibody was coupled to an Alexa488 (Millipore) fluorescent dye. TUNEL staining was performed using the Apoptosis Detection Kit, Millipore (Lot 3192170). In immunohistochemical stainings and BrdU incorporation assays, nuclei were counterstained with 4′,6-diamidin-2-phenylindole (DAPI). Antibodies and reagents were also used for immunocytochemical staining of dissociated mammary glands at a 2–fivefold higher dilution. Samples were usually documented with a Leica DMI 6000B inverted microscope (Leica) equipped with a DFC 360FX camera (Leica). For confocal images, a Leica TCS SL confocal microscope was used.

### Histological stainings

For whole-mount carmine-alum staining, paraformaldehyde-fixed mammary glands (n = 3–7 per condition) were submerged in Carnoy’s solution (3 parts ethanol, 1 part glacial acetic acid) for 18–24 h before staining overnight with carmine-alum (Roth) and transferral in destaining solution (500 ml 70% ethanol, 1.25 ml 25% HCl). Destaining solution was changed every 30 min until it stayed clear. Afterwards, glands were dehydrated in an ascending alcohol series before clearing with benzyl alcohol/benzyl benzoate (1:2). For examination of YFP fluorescence in *MMTV::Cre(A) Rosa26*^*stopfloxYFP*^ or *MMTV::Cre(F) Rosa26*^*stopfloxYFP*^ mice and carmine-alum whole-mounts, mammary glands were spread between two glass slides before imaging. For histological examination mammary glands were fixed in 4% paraformaldehyde, embedded in paraffin, sectioned at 10 µm with a Leica RM2165 rotary microtome and stained with Mayer's hemalum solution (Merck) and Eosin G (Merck). Oil red O staining (Sigma) was performed on 10 µm mammary gland cryostat sections. Sections were stained with a 0.5% Oil red O (Sigma) in isopropanol solution for 15 min and counterstained with Mayer's hemalum.

X-gal stainings on embryonic ventral dermis and mammary glands followed standard procedures. Samples were analyzed and documented either with a Leica MZFLIII stereomicroscope equipped with an Axiocam (Zeiss) or a with a Leica DMR microscope equipped with a Leica DFC420 C camera.

### SDS page, Coomassie staining, determination of protein and lipid content

Milk was collected during the second week of lactation at every second day for three times and equal volumes were combined for determination of milk parameters from each dam. Milk proteins were size-fractionated on polyacrylamide-SDS gels, and stained with Coomassie Brilliant Blue G 250 (Serva). Total milk protein content was determined with the Pierce BCA Protein Assay Kit (Thermofisher) on a 1:100 milk in water dilution. The amount of milk triglycerides was analyzed by photometric determination of the amount of oxidized NADH at 340 nm in a reaction containing milk triglycerides, phosphoenolpyruvate, ATP, lipase, glycerol kinase, pyruvate kinase, lactate dehydrogenase and NADH.

### DNA constructs

Luciferase reporter plasmids carrying the promoter of the miR-424(322)/503 cluster or parts of it, were generated by PCR-amplification of sequences from human genomic DNA (ChrX: 134546544-134550090 according to hg19) and insertion into the pGL2 luciferase vector (Promega). Site-directed mutagenesis of Sox10 binding sites in the core promoter fragment was performed with the Q5 Site-Directed Mutagenesis Kit (New England Biolabs). Expression plasmids for full length Sox10 were based on pCMV5^45,46^. The Sox10 expressing retrovirus was as described^47^.

### Cell culture, transfection, extract preparation, electrophoretic mobility shift analysis (EMSA), and luciferase assays

Rat Oln93 cells (RRID:CVCL_5850), mouse N2a (ATCC Cat# CCL-131, RRID:CVCL_0470) and human HEK293 cells (ATCC Cat# CRL-1573, RRID:CVCL_0045) were maintained in DMEM containing 10% FCS. Mouse HC11 cells (ATCC Cat# CRL-3062, RRID:CVCL_0288) were maintained in RPMI medium containing 10% FCS, 10 mM HEPES, 5 µg/ml insulin and 10 ng/ml epidermal growth factor (EGF). Lactogenic differentiation of HC11 cells was performed as described^22^. Briefly, cells were grown to confluence. Then cells were kept for a day in medium without EGF and for another 6 days in medium containing 1 µM dexamethasone and 5 µg/ml prolactin instead of EGF before harvest.

HEK293 cells were transfected by polyethylenimine using 10 µg pCMV empty or pCMV-Sox10 expression plasmid per 100-mm plate. Cells were harvested 48 h post-transfection for extract preparation^45^.

EMSA was performed using ^32^P-labeled double-stranded oligonucleotides containing putative Sox10 binding sites, poly-dGdC as unspecific competitor and HEK293 whole cell extracts as protein source^45^. Oligonucleotides were 22–24 bp in length (for sequences see Fig. 8g).

For luciferase reporter assays, N2a cells were transfected in triplets using SuperFect Reagent (Qiagen) with 0.1 µg luciferase reporter plasmid in combination with 0.15 µg pCMV5-based expression plasmids per well of a 24-well plate. Whole cell extracts were prepared, and luciferase activities determined in the presence of luciferin and ATP 48 h posttransfection. Sox10-dependent fold activations ± SEM were calculated by arbitrarily setting the luciferase activity in the absence of Sox10 to 1 and putting the activities in the presence of Sox10 in relation to it.

### RNA isolation, quantitative RT-PCR (qRT-PCR)

Total RNA was prepared from Oln93, differentiated HC11 cells, whole mouse mammary glands and FACS-purified mammary epithelial cells using the miRVana miRNA isolation kit (Ambion, ThermoFisher). For expression studies on miR-424 and miR-503, target-specific cDNA synthesis and individual TaqMan MicroRNA Assays (assay ID 001076 for miR-424, 002456 for miR-503) were performed on a Bio-Rad CFX96 Real-Time PCR system. Expression levels were normalized to *U6* snRNA, set arbitrarily to 1 for each microRNA in the control and expressed relative to it for altered cells and tissues.

### Chromatin immunoprecipitation

Chromatin was prepared from dissociated cells of mammary glands 6 h after weaning following treatment with 1% paraformaldehyde, quenching with 0.125 M glycine and shearing to 200–400 bp fragments. After pre-clearing, chromatin was incubated with rabbit antisera against Sox10 and corresponding preimmune antiserum before addition of protein A sepharose beads and precipitation. After crosslink reversal, proteinase K treatment and purification of DNA from input and precipitated chromatin, the following primer pairs were used in quantitative PCRs on a Bio-Rad CFX96 thermocycler with each reaction performed in triplicates: 5′-ACTAATGACGAGGTGATTACTGT-3′ and 5′-GTGAAACTTCCTGCTACCTTGT-3′ (amplifying ChrX: 53054563-53054765 according to mm10, corresponding to the miR-424(322)/503 promoter), 5′-GGAACTTAAGCCACGCCTTG-3′ and 5′-GGGAAGTTGGTGAGGGAGAA-3′ (amplifying ChrX: 53052684-53052889 according to mm10, corresponding to a control genomic region). The ΔΔCt method was used to calculate the recovery rates of a given DNA segment. Recovery rates for preimmune serum were set to 1 and those for the anti-Sox10 antiserum set in relation to it.

### Quantification and statistical analysis

For quantification sections or dissociated single cell preparations of mammary glands from at least three independent mice per genotype were counted. Typical bulbous structures at the end of the ducts were counted as TEBs. End points without bulbous structures were quantified as ductal tips. Quantification of fluorescence intensity was performed using NIH ImageJ software as described^48^. All statistical analysis was performed with Prism6 software (GraphPad) or Excel 2016 (Microsoft Office). Statistically significant differences were determined by Student’s t test or One-way ANOVA with Bonferroni’s multiple comparisons test as indicated in the figure legends.

### Ethics statement

Mice experiments were in accord with animal welfare laws and approved by the responsible local ethics committees and government bodies (University, Veterinäramt Stadt Erlangen & Regierung von Unterfranken, TS-00/12 Biochemie II).

## Supplementary information

Supplementary information

## Data Availability

All data generated or analyzed during this study are included in this published article and its supplementary information files.

## References

[1] Wegner M (1999). From head to toes: the multiple facets of Sox proteins. Nucleic Acids Res..

[2] Kelsh RN (2006). Sorting out Sox10 functions in neural crest development. BioEssays.

[3] Weider M, Wegner M (2017). SoxE factors: transcriptional regulators of neural differentiation and nervous system development. Semin. Cell Dev. Biol..

[4] Britsch S (2001). The transcription factor Sox10 is a key regulator of peripheral glial development. Genes Dev..

[5] Cheung M (2005). The transcriptional control of trunk neural crest induction, survival, and delamination. Dev. Cell.

[6] Finzsch M (2010). Sox10 is required for Schwann cell identity and progression beyond the immature Schwann cell stage. J. Cell Biol..

[7] Stolt CC (2002). Terminal differentiation of myelin-forming oligodendrocytes depends on the transcription factor Sox10. Genes Dev..

[8] Reiprich S, Stolt CC, Schreiner S, Parlato R, Wegner M (2008). SoxE proteins are differentially required in mouse adrenal gland development. Mol. Biol. Cell.

[9] Dravis C (2015). Sox10 regulates stem/progenitor and mesenchymal cell states in mammary epithelial cells. Cell Rep..

[10] Lyons WR (1958). Hormonal synergism in mammary growth. Proc. R. Soc. Lond. B Biol. Sci..

[11] Nandi S (1958). Endocrine control of mammarygland development and function in the C3H/ He Crgl mouse. J. Natl. Cancer Inst..

[12] Inman JL, Robertson C, Mott JD, Bissell MJ (2015). Mammary gland development: cell fate specification, stem cells and the microenvironment. Development.

[13] Cowin P, Wysolmerski J (2010). Molecular mechanisms guiding embryonic mammary gland development. Cold Spring Harb. Perspect. Biol..

[14] Dravis C (2018). Epigenetic and transcriptomic profiling of mammary gland development and tumor models disclose regulators of cell state plasticity. Cancer Cell.

[15] Cimino-Mathews A (2013). Neural crest transcription factor Sox10 is preferentially expressed in triple-negative and metaplastic breast carcinomas. Hum. Pathol..

[16] Werner T, Hammer A, Wahlbuhl M, Bösl MR, Wegner M (2007). Multiple conserved regulatory elements with overlapping functions determine Sox10 expression in mouse embryogenesis. Nucleic Acids Res..

[17] Wagner KU (1997). Cre-mediated gene deletion in the mammary gland. Nucleic Acids Res..

[18] Wagner KU (2001). Spatial and temporal expression of the Cre gene under the control of the MMTV-LTR in different lines of transgenic mice. Transgenic Res..

[19] Wegner M (2010). All purpose Sox: the many roles of Sox proteins in gene expression. Int. J. Biochem. Cell Biol..

[20] Llobet-Navas D (2014). The miR-424(322)/503 cluster orchestrates remodeling of the epithelium in the involuting mammary gland. Genes Dev..

[21] Reiprich S (2017). Transcription factor Sox10 regulates oligodendroglial Sox9 levels via microRNAs. Glia.

[22] Morrison B, Cutler ML (2009). Mouse mammary epithelial cells form mammospheres during lactogenic differentiation. J. Vis. Exp..

[23] Malhotra GK (2014). The role of Sox9 in mouse mammary gland development and maintenance of mammary stem and luminal progenitor cells. BMC Dev. Biol..

[24] Gu B (2009). Pygo2 expands mammary progenitor cells by facilitating histone H3 K4 methylation. J. Cell Biol..

[25] Lindvall C (2009). The Wnt co-receptor Lrp6 is required for normal mouse mammary gland development. PLoS ONE.

[26] Herbarth B (1998). Mutation of the Sry-related Sox10 gene in *Dominant megacolon*, a mouse model for human Hirschsprung disease. Proc. Natl. Acad. Sci. USA.

[27] Inoue K (2004). Molecular mechanism for distinct neurological phenotypes conveyed by allelic truncating mutations. Nat. Genet..

[28] Pingault V (1998). Sox10 mutations in patients with Waardenburg-Hirschsprung disease. Nat. Genet..

[29] Southard-Smith EM, Kos L, Pavan WJ (1998). Sox10 mutation disrupts neural crest development in *Dom* Hirschsprung mouse model. Nat. Genet..

[30] Bach K (2017). Differentiation dynamics of mammary epithelial cells revealed by single-cell RNA sequencing. Nat. Commun..

[31] Bremer M (2011). Sox10 is required for Schwann cell homeostasis and myelin maintenance in the adult peripheral nerve. Glia.

[32] Fröb F (2012). Establishment of myelinating Schwann cells and barrier integrity between central and peripheral nervous systems depend on Sox10. Glia.

[33] Aprato J (2020). Myrf guides target gene selection of transcription factor Sox10 during oligodendroglial development. Nucleic Acids Res..

[34] Hornig J (2013). The transcription factors Sox10 and Myrf define an essential regulatory network module in differentiating oligodendrocytes. PLoS Genet..

[35] Guo W (2012). Slug and Sox9 cooperatively determine the mammary stem cell state. Cell.

[36] Kogata N (2018). Sox9 regulates cell state and activity of embryonic mouse mammary progenitor cells. Commun. Biol..

[37] Finzsch M, Stolt CC, Lommes P, Wegner M (2008). Sox9 and Sox10 influence survival and migration of oligodendrocyte precursors in the spinal cord by regulating PDGF receptor alpha expression. Development.

[38] Shakhova O (2015). Antagonistic cross-regulation between Sox9 and Sox10 controls an anti-tumorigenic program in melanoma. PLoS Genet..

[39] Srinivas S (2001). Cre reporter strains produced by targeted insertion of EYFP and ECFP into the ROSA26 locus. BMC Dev. Biol..

[40] Mao X, Fujiwara Y, Orkin SH (1999). Improved reporter strain for monitoring Cre recombinase-mediated DNA excisions in mice. Proc. Natl. Acad. Sci. USA.

[41] Madisen L (2010). A robust and high-throughput Cre reporting and characterization system for the whole mouse brain. Nat. Neurosci..

[42] Matsuoka T (2005). Neural crest origins of the neck and shoulder. Nature.

[43] Muranishi Y (2016). Method for collecting mouse milk without exogenous oxytocin stimulation. Biotechniques.

[44] Maka M, Stolt CC, Wegner M (2005). Identification of Sox8 as a modifier gene in a mouse model of Hirschsprung disease reveals underlying molecular defect. Dev. Biol..

[45] Kuhlbrodt K, Herbarth B, Sock E, Hermans-Borgmeyer I, Wegner M (1998). Sox10, a novel transcriptional modulator in glial cells. J. Neurosci..

[46] Kuhlbrodt K (1998). Functional analysis of Sox10 mutations found in human Waardenburg-Hirschsprung patients. J. Biol. Chem..

[47] Braun SM (2015). Programming hippocampal neural stem/progenitor cells into oligodendrocytes enhances remyelination in the adult brain after injury. Cell Rep..

[48] McCloy RA (2014). Partial inhibition of Cdk1 in G 2 phase overrides the SAC and decouples mitotic events. Cell Cycle.

