# Supplementary material for: The transcription factor Sox10 is an essential determinant of branching morphogenesis and involution in the mouse mammary gland
Source: Sci Rep. 2020 Oct 20;10:17807. doi: 10.1038/s41598-020-74664-y (PMC7575560; doi:10.1038/s41598-020-74664-y)
Supplement: Supplementary file 1 — Supplementary information [file 41598_2020_74664_MOESM1_ESM.doc]

Supplementary Information

for

The transcription factor Sox10 is an essential determinant of branching morphogenesis and involution in the mouse mammary gland

by

Svenja Mertelmeyer, Matthias Weider, Tina Baroti, Simone Reiprich, Franziska Fröb, C. Claus Stolt, Kay-Uwe Wagner and Michael Wegner


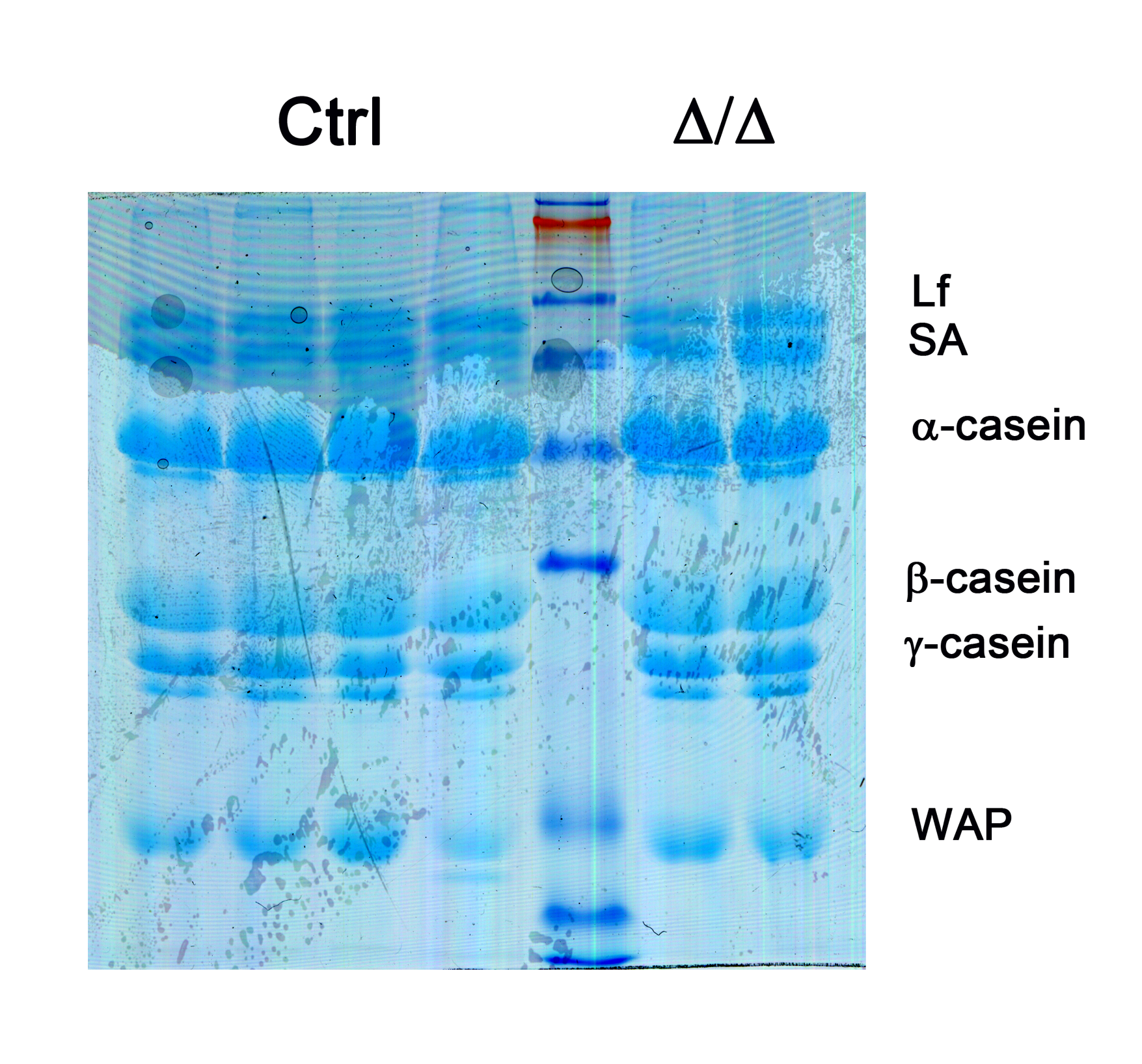


**Supplementary Figure 1: Full Coomassie blue-stained SDS polyacrylamide gel for Fig.6e** after electrophoresis of milk proteins from lactating adult control (Ctrl) and *Sox10 macko* (Δ/Δ) mice. Size marker is between the four control and two Δ/Δ samples. Lf, lactoferrin; SA, serum albumin; WAP, whey acid protein.
